# Genetic interrelationships of *Spirometra erinaceieuropaei* (Cestoda: Diphyllobothriidea), the causative agent of sparganosis in Europe

**DOI:** 10.1051/parasite/2022009

**Published:** 2022-02-11

**Authors:** Eva Čisovská Bazsalovicsová, Alžbeta Radačovská, Antti Lavikainen, Roman Kuchta, Ivica Králová-Hromadová

**Affiliations:** 1 Institute of Parasitology, Slovak Academy of Sciences Hlinkova 3 040 01 Košice Slovakia; 2 Department of Veterinary Biosciences (Veterinary Parasitology), Faculty of Veterinary Medicine, University of Helsinki Agnes Sjöberginkatu 2 Helsinki 00014 Finland; 3 Institute of Parasitology, Biology Centre, Czech Academy of Sciences Branišovská 31 370 05 České Budějovice Czech Republic

**Keywords:** Food/water-borne zoonosis, Sparganosis in Finland, *cox*1 haplotypes, Molecular genotyping, Genetic lineages

## Abstract

The geographic distribution of *Spirometra erinaceieuropaei* (Cestoda: Diphyllobothriidea), the causative agent of food/water-borne sparganosis, is restricted to Europe, where infected canids, felids, mustelids, suids, and reptiles have been documented from Poland, Ukraine, Belarus, Russia, Serbia, Estonia, Latvia, and Finland. The main objective of the current study was to map the molecular divergence of *S. erinaceieuropaei* from Finland using the complete sequences of the mitochondrial cytochrome *c* oxidase subunit 1 gene (*cox*1 mtDNA). Seven *cox*1 haplotypes were determined in 15 tapeworms from Eurasian lynx (*Lynx lynx*) from three localities in southern Finland. In addition, the first inter-population study of *S. erinaceieuropaei* based on currently obtained data on *cox*1 from Finland and previously published data from Finland, Latvia, Ukraine, and Poland, was performed. The haplotype network showed a star-like pattern without specific subdivision of lineages according to the locality. Samples from Finland, Latvia, and Poland shared several haplotypes and formed the common Baltic lineage. The haplotype of *S. erinaceieuropaei* from Ukraine was unique and placed on a separate mutational pathway, suggesting a different lineage of the parasite.

## Abstract (fr)

La distribution géographique de *Spirometra erinaceieuropaei* (Cestoda : Diphyllobothriidea), l’agent causal de la sparganose d’origine alimentaire/hydrique, est limitée à l’Europe, où des canidés, félidés, mustélidés, suidés et reptiles infectés ont été documentés en Pologne, Ukraine, Biélorussie, Russie, Serbie, Estonie, Lettonie et Finlande. L’objectif principal de la présente étude était de cartographier la divergence moléculaire de *S. erinaceieuropaei* de Finlande à l’aide des séquences complètes du gène mitochondrial de la sous-unité 1 de la cytochrome c oxydase (ADNmt *cox1*). Sept haplotypes *cox1* ont été déterminés chez quinze cestodes du Lynx d’Eurasie (*Lynx lynx*) de trois localités du sud de la Finlande. En outre, la première étude inter-populationnelle de *S. erinaceieuropaei* basée sur les données actuellement obtenues sur *cox1* de Finlande et sur des données précédemment publiées de Finlande, Lettonie, Ukraine et Pologne, a été réalisée. Le réseau d’haplotypes a montré un motif en étoile sans subdivision spécifique des lignées selon la localité. Des échantillons de Finlande, Lettonie et Pologne partagent plusieurs haplotypes et forment la lignée commune de la Baltique. L’haplotype de *S. erinaceieuropaei* d’Ukraine est unique et placé sur une voie de mutation distincte suggérant une lignée différente du parasite.

## Introduction

Sparganosis is a food- and water-borne parasitic zoonosis caused by tapeworm larvae of the genus *Spirometra* Faust, Campbell & Kellogg, 1929 (Cestoda: Diphyllobothriidea). The first larval stage (procercoid) of these tapeworms develops in the first intermediate hosts, copepods (*Cyclops* sp.). The second intermediate or paratenic hosts are many wild and domesticated vertebrates, including amphibians, reptiles, birds, and mammals, in which plerocercoids (spargana) develop mainly in subcutaneous tissues, but also in musculature and internal organs [14]. The definitive hosts are wild and domesticated carnivores. Humans become infected either by drinking water containing infected copepods or by consuming plerocercoids in raw or inadequately cooked meat of the second intermediate or paratenic host [9, 14].

Taxonomy of the genus *Spirometra* has always been complicated due to high intraspecific variability, uniformity of most diphyllobothriid taxa, and lack of reliable species-specific morphological markers [14]. A recent phylogenetic analysis of members of the genus *Spirometra* based on the sequences of the mitochondrial cytochrome *c* oxidase subunit 1 gene (*cox*1 mtDNA) revealed the presence of six molecularly well-defined and geographically distinct lineages (lin.) corresponding to separate species, namely *Spirometra erinaceieuropaei* (Rudolphi 1819) (European lin.), *Spirometra decipiens* (Diesing, 1850) complex 1 (American lin. 1), *Spirometra decipiens* complex 2 (American lin. 2), *Spirometra folium* (Diesing, 1850) (African lin.), *Spirometra mansoni* (Cobbold, 1883) (Eurasian, Oceanic, and African lin.), and *Spirometra* sp. 1 (Asian lin. 2) [14].

The type species *S. erinaceieuropaei* (syn. *Spirometra erinacei*) was described more than 200 years ago on the basis of plerocercoids found in the European hedgehog (*Erinaceus europaeus*) at an unknown locality (probably Brandenburg or former Prussia) in Europe [19]. The geographic distribution of this species was long considered cosmopolitan, because it was misidentified with the widespread but genetically distinct *S. mansoni*, which occurs in East and Southeast Asia, namely China, Thailand, Vietnam, Laos, Myanmar, India, Indonesia, Japan, and also Australia [14]. Even though a recent comprehensive phylogenetic study revealed that Asian and Australian *Spirometra* specimens are related to *S. mansoni*, its occurrence was surprisingly confirmed by molecular methods from edible frogs (*Pelophylax esculentus*) in Romania (south-eastern Europe). On the other hand, *S. erinaceieuropaei* was found to be restricted exclusively to north-eastern Europe [14].

In the past two decades, the occurrence of *S. erinaceieuropaei* in wild carnivores, mustelids, wild boars, and snakes has been reported in several northern and eastern European countries, mainly in the vicinity of the Białowieża National Park in eastern Poland [4–9, 14] and in western Belarus [22, 24, 25], but also in Serbia [17], Estonia [10], Latvia [1], Ukraine, and Finland [11, 14, 15]. The genetic structure of *S. erinaceieuropaei* based on the mitochondrial *cox*1 gene is so far available only for the populations from Poland and Latvia [1, 7] and only single sequences were published for tapeworms from Finland and Ukraine [14, 15].

The main objective of the current study was to evaluate, for the first time, genetic interrelationships among different European populations of *S. erinaceieuropaei* using complete *cox*1 sequences. Since only three specimens from Finland have been sequenced so far, additional isolates from European lynx (*Lynx lynx*) from three localities in Finland were used to obtain a broader dataset on *S. erinaceieuropaei* from this northernmost locality of its occurrence.

## Materials and methods

Fifteen immature and adult tapeworms were isolated from three Eurasian lynx from southern Finland in 2008 and 2010. A single tapeworm was obtained from a lynx that was legally hunted near Metsäkylä village in Hamina municipality, another specimen originated from a dead lynx found in Savonlinna municipality, and 13 tapeworms were isolated from a lynx near Kallasti farm in Ylämaa municipality, which was euthanized due to severe injury. Material was provided by the Natural Resources Institute and the Finnish Food Safety Authority EVIRA with special permissions of hunting rules and regulations, following ethics guidelines. Tapeworms were washed in saline solution and preserved in 70% ethanol for further analysis.

All specimens were initially identified as diphyllobothriid tapeworms (genera *Dibothriocephalus* and/or *Spirometra*) based on their external morphological characteristics. Taxonomic identification of diphyllobothriids is quite complicated due to the numerous unstable and overlapping morphological characters of each taxon, making the definition of species boundaries difficult or even impossible [14]. Moreover, tapeworms recovered from *post mortem* hosts were either immature or even fragmented and decomposed, making correct identification impossible [13, 21]. Therefore, accurate taxonomic determination must rely on species-specific molecular markers.

Genomic DNA was isolated from 20 mg of tissue of the distal part of tapeworm using a QIAamp^®^ DNA Kit (QIAGEN, Hilden, Germany), according to the manufacturer’s instructions, diluted in deionized water and stored at −20 °C. Details of PCR amplification, sequencing, sequence assembly and sequence analyses were published previously [1]. The anterior part of each tapeworm was stained and mounted on microscopic slides as morphological voucher and was deposited in the Helminthological Collection of the Institute of Parasitology, Biology Centre of the Czech Academy of Sciences, České Budějovice, Czech Republic (IPCAS No. C-101).

The initial molecular genotyping was based on the partial sequences (640 bp) of the large subunit of the ribosomal RNA gene (*lsr*DNA). The aim was to distinguish between the genera *Spirometra* and *Dibothriocephalus*, which could infect Eurasian lynx in Finland. PCR amplification of the *lsr*DNA was performed using the forward primer LSU-5 (5′–TAGGTCGACCCGCTGAAYTTAAGCA–3′) and the reverse primer 1500R (5′–GCTATCCTGAGGGAAACTTCG–3′) [18]. The 5′-end of the *lsr*DNA was sequenced from both directions using the LSU-5 primer and two internal primers, 300F (5′–CAAGTACCGTGAGGGAAAGTTG–3′) and 400R (5′–GCAGCTTGACTACACCCG–3′) [18]. Fourteen of the fifteen tapeworms had identical *lsr*DNA sequence structure, and the *lsr*DNA of one specimen from Kallasti differed by one mutation (99.8% pairwise sequence identity). The sequences were deposited in the GenBank, EMBL and DDBJ databases under accession numbers MW365689–MW365702. They showed 99.8–100% similarity/identity with the *lsr*DNA of *S. erinaceieuropaei* from Eurasian lynx (MT313931) and grey wolf (MT321262) from Latvia [1].

To confirm these results, all tapeworms were also analysed using complete mitochondrial *cox*1, which has been showed to be the most reliable identification tool for diphyllobothriids [14]. PCR amplification and sequencing were performed using the primers Diphyllo-Cox1-F (5′–TAGACTAAGTGTTTTCAAAACACTA–3′) and Diphyllo-Cox1-R (5′–ATAGCATGATGCAAAAGG–3′) [27]. Two internal primers, DCox1-R2 (5′–AAACACCGGCTCACGTAAAG–3′) and Cox1-R3 (5′–CGCAAATGCCGAATAAAGAG–3′) [7] were used to sequence the complete *cox*1 from both directions. The sequences were deposited in the GenBank, EMBL and DDBJ databases under accession numbers MW357422, MW357425–MW357433, MW357435–MW357437, and MW357539–MW357540.

**Figure 1:**
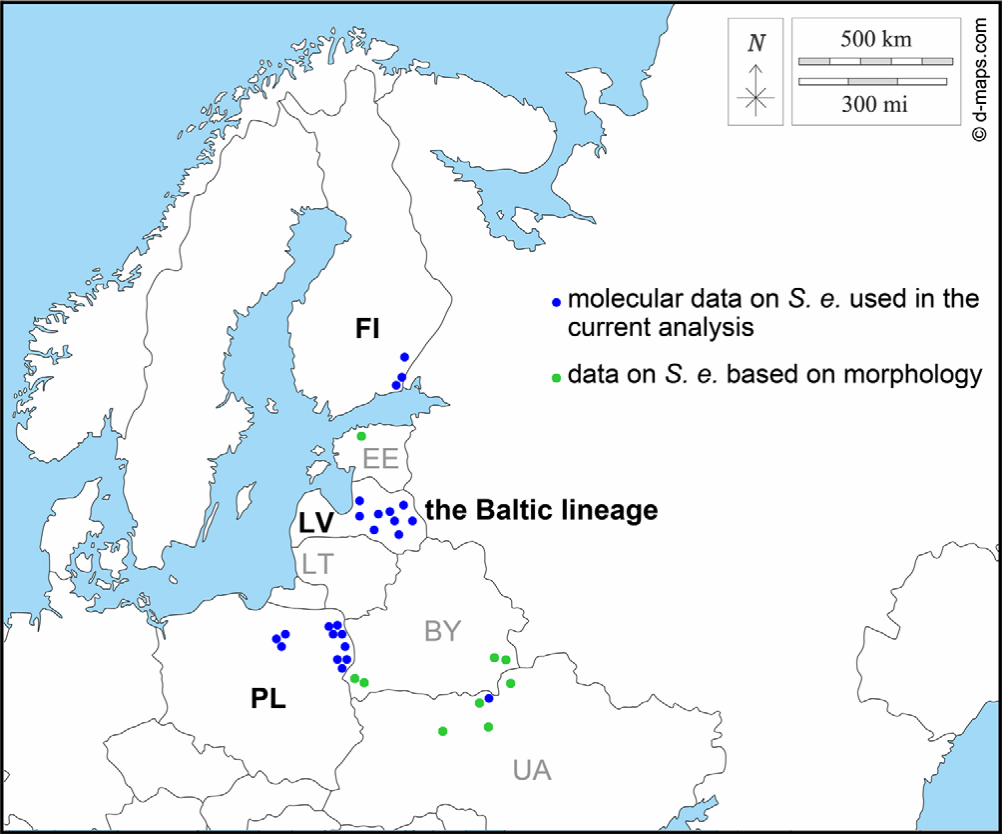
Scheme of the distribution of *Spirometra erinaceieuropaei* (*S. e*.) from Finland (FI), Poland (PL), Latvia (LV), and Ukraine (UA) analysed molecularly in the current work (blue points) and its findings from neighbouring Estonia (EE), Belarus (BY), and Ukraine (UA) based on its morphology (green points). The map was obtained from https://d-maps.com.

The newly obtained data from 15 *S. erinaceieuropaei* from Finland were used in the study of genetic interrelationships among populations together with the previously published data, namely: (i) 50 *cox*1 haplotypes (Ha1–Ha50; MK523394–MK523443 and MT131826–MT131829) detected in 319 individuals from canids, mustelids, and reptiles from different localities in north-eastern Poland [7, 14]; (ii) 12 *cox*1 haplotypes (CO1-Ha1-Ha12/LV; MT941768–MT941770 and MT951146–MT951155) detected in three individuals from Eurasian lynx and nine specimens of grey wolf (*Canis lupus*) from Latvia [1]; (iii) one haplotype (MT131830) detected in a single tapeworm from grey wolf from Chernobyl in Ukraine [14]; and (iv) one haplotype (MT131825) of single tapeworm from Eurasian lynx from Kallasti in Finland [14] (Fig. 1, blue points). Genetic variability among populations from four European countries was visualised by a haplotype network using PopArt [16] with the TCS 1.21 algorithm [2].

## Results

Analysis of the complete *cox*1 sequences (1566 bp) of 15 *S. erinaceieuropaei* specimens from Finland revealed a high degree of sequence similarity (99.6–99.9%) within the population. Seven *cox*1 haplotypes CO1–Ha1–Ha7/FI were determined; haplotypes CO1–Ha2/FI and CO1–Ha4/FI were each detected in a single tapeworm, while haplotypes CO1–Ha5–7/FI were each shared by two individuals. Three tapeworms possessed CO1–Ha3/FI and four individuals were characterised by CO1–Ha1/FI.

**Figure 2:**
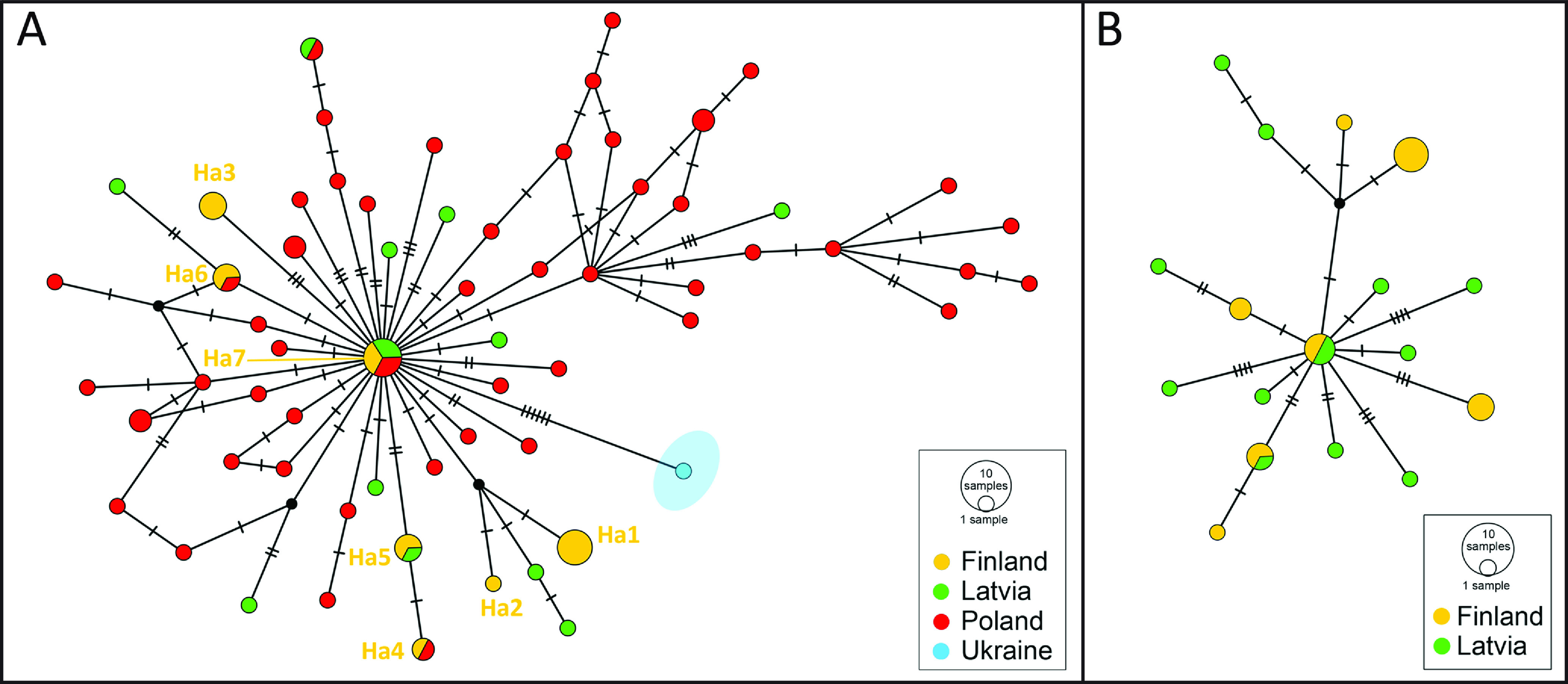
Haplotype network diagram based on mitochondrial *cox*1 haplotypes of *Spirometra erinaceieuropaei* from: (A) Finland (current data), Latvia [1], Poland [7, 14], and Ukraine [14]; and (B) specifically for Finland and Latvia. The sizes of haplotypes are proportional to the number of samples. Codes denote haplotype identifiers detected in individuals from Finland. Each hatch mark represents a single mutation, while black dots symbolise intermediate missing or unsampled haplotypes.

The haplotype network based on the complete *cox*1 sequences showed a star-like pattern and provided pilot information on the interrelationships of *S. erinaceieuropaei* from north-eastern Europe (Fig. 2A). The entire dataset contained 70 variable characters, 33 of which were parsimony informative. No specific subdivision of lineages depending on locality was detected. Several haplotypes, including the central haplotype, were shared by *S. erinaceieuropaei* from Finland, Latvia and Poland, countries of the Baltic region. The tapeworms from Finland and Latvia shared one haplotype, while specimens from Finland and Poland shared two haplotypes (Fig. 2A). This suggests that *S. erinaceieuropaei* from Finland, Latvia and Poland form a common group referred to as “the Baltic lineage”. In contrast, the single haplotype of *S. erinaceieuropaei* from Ukraine was on a separate mutational pathway displayed by six substitutions (Fig. 2A), indicating a genetic distance between the tapeworms from Ukraine and the Baltic lineage. Datasets from Finland and Latvia were analysed separately in an individual haplotype network, confirming an overlapping genetic structure of *S. erinaceieuropaei* from these two countries (Fig. 2B).

## Discussion

The current study provided pilot data on the genetic interrelationships of European populations of *S. erinaceieuropaei* and opened several perspectives for future studies. One of the challenges is to determine the geographic boundaries of the Baltic lineage. In addition to Finland, Latvia, and Poland, tapeworms morphologically identified as *S.* cf. *erinaceieuropaei* were also detected in a stray cat from Estonia [10] and in several mammals in south-western Belarus [23, 24, 25, 26] (Fig. 1, green points). Moreover, the white spot on the map of the north-eastern distribution of *S. erinaceieuropaei* is Lithuania (Fig. 1, LT), the only Baltic country for which no data on this tapeworm are available. Screening and molecular genotyping of *S. erinaceieuropaei* from the above mentioned countries would provide a more complex picture of its geographic distribution in north-eastern Europe and better specification of the Baltic lineage.

Haplotype diversity detected in the current work decreased from the southern geographical regions towards the northern ones. The highest number of haplotypes (50) was reported from Poland [7], followed by 12 haplotypes in Latvia [1] and seven in Finland (current work). However, these data are based on a rather unequal number of analysed samples (319/Poland; 13/Latvia; 15/Finland) and require further studies supplemented with more material.

The specific *cox*1 haplotype of a tapeworm from Ukraine provided the preliminary evidence for a different genetic structure of *S. erinaceieuropaei* from Eastern Europe. Since the data are based on only one specimen, no decisive conclusions can be drawn about genetic interrelationships between the tapeworms from Ukraine and the Baltic region. It is obvious that molecular analysis based on a broader set of samples is necessary to determine the diversity of *Spirometra* from different Eastern European countries. In particular, *S.* cf. *erinaceieuropaei* was morphologically confirmed in wildlife in the northern part of Ukraine [11], in the Białowieża Forest and Biarezinski Biosphere Reserve in Belarus [23, 24, 25, 26] (Fig. 1, green points), and in several localities in Russia (e.g. Tver, Moscow region and Astrakhan Reserve) [3, 12].

The numerous findings of *S. erinaceieuropaei* in wildlife from different European regions acquired after the year 2000 have demonstrated the importance of ongoing screening of the causative agents of sparganosis for a better understanding of their current distribution in Europe. Reliable molecular markers must be applied to determine whether south-eastern Europe is a region with exclusive presence of *S. mansoni*, or a place with sympatric occurrence of *S. mansoni* and *S. erinaceieuropaei.* Their overlapping distribution cannot be ruled out since the latter species has been confirmed morphologically in wild boars in Serbia [17, 20] and in forest cats from the Odesa region of south-eastern Ukraine [11]. It is now evident that a multidisciplinary approach using morphological, biological and molecular methods needs to be applied for accurate determination of spatial distribution of *S. erinaceieuropaei* and *S. mansoni* in Europe and an assessment of the interrelationships among their populations.

## Conflict of interest

The authors declare that they have no conflict of interest.
